# Effect of phenolic acids of microbial origin on production of reactive oxygen species in mitochondria and neutrophils

**DOI:** 10.1186/1423-0127-19-89

**Published:** 2012-10-12

**Authors:** Natalia Beloborodova, Iskander Bairamov, Andrei Olenin, Victoria Shubina, Vera Teplova, Nadezhda Fedotcheva

**Affiliations:** 1 Negovsky Research Institute of General Reanimatology Russian Academy of Medical Sciences, Laboratory of Metabolism of Critical State, Moscow, Russia; 2 Department of Chemistry, Lomonosov Moscow State University, Moscow, Russia; 3 Institute of Theoretical and Experimental Biophysics, Russian Academy of Sciences, Pushchino, Moscow region, Russia

**Keywords:** Sepsis, Phenolic acids, Mitochondria, Neutrophils, Reactive oxygen species

## Abstract

**Background:**

Several low-molecular-weight phenolic acids are present in the blood of septic patients at high levels. The microbial origin of the most of phenolic acids in the human body was shown previously, but pathophysiological role of the phenolic acids is not clear. Sepsis is associated with the excessive production of reactive oxygen species (ROS) in both the circulation and the affected organs. In this work the influence of phenolic acids on ROS production in mitochondria and neutrophils was investigated.

**Methods:**

ROS production in mitochondria and neutrophils was determined by MCLA- and luminol-dependent chemiluminescence. The rate of oxygen consumption by mitochondria was determined polarographically. The difference of electric potentials on the inner mitochondrial membrane was registered using a TPP^+^-selective electrode. The formation of phenolic metabolites in monocultures by the members of the main groups of the anaerobic human microflora and aerobic pathogenic bacteria was investigated by the method of gas chromatography–mass spectrometry.

**Results:**

All phenolic acids had impact on mitochondria and neutrophils, the main producers of ROS in tissues and circulation. Phenolic acids (benzoic and cinnamic acids) producing the pro-oxidant effect on mitochondria inhibited ROS formation in neutrophils. Their effect on mitochondria was abolished by dithiothreitol (DTT). Phenyllactate and p-hydroxyphenyllactate decreased ROS production in both mitochondria and neutrophils. Bifidobacteria and lactobacilli produced in vitro considerable amounts of phenyllactic and *p*-hydroxyphenyllactic acids, *Clostridia s.* produced great quantities of phenylpropionic and *p*-hydroxyphenylpropionic acids, p-hydroxyphenylacetic acid was produced by *Pseudomonas aeruginosa* and *Acinetobacter baumanii*; and benzoic acid, by *Serratia marcescens*.

**Conclusions:**

The most potent activators of ROS production in mitochondria are phenolic acids whose effect is mediated via the interaction with thiol groups. Among these are benzoic and cinnamic acids. Some phenolic acids, in particular phenyllactate and p-hydroxyphenyllactate, which decrease ROS production in mitochondria and neutrophils, can play a role of natural antioxidants. The results indicate that low-molecular weight phenolic acids of microbial origin participate in the regulation of the ROS production in both the circulation and tissues, thereby affecting the level of oxidative stress in sepsis.

## Background

The systemic inflammatory response syndrome (SIRS) is the most frequent complication in critical states, which develop as a result of severe diseases, traumata, and sepsis. The development of SIRS is a complex process, which is accompanied by hematological, hemodynamic, and metabolic disorders [1-3]. Two stages of SIRS are recognized: the initial proinflammatory stage, which involves the activation of neutrophils, tissue macrophages, an increase in the production of cytokines and reactive oxygen species (ROS), and the later stage, which is characterized by a decrease in the level of cytokines and neutrophils, the appearance of the signs of multiple organ failure and persistent tissue hypoxia [4]. The mechanisms of the development of multiple organ failure remain still obscure. The prevalent hypothesis regarding the mechanisms of sepsis and septic shock indicates that this syndrome is caused by an excessive defensive and inflammatory responses characterized by an increased generation of ROS, nitric oxide (NO), and inflammatory cytokines [4,5]. It was assumed that the level of oxidative stress is crucial in the genesis and outcome of sepsis [6]. Sepsis is associated with the excessive ROS production in both the circulation and the affected organs. In pathological complications, such as acute lung injury and sepsis, excess ROS production by neutrophils may influence vicinal cells of endothelium or epithelium, contributing to the inflammatory tissue injury [7,8].

Along with neutrophils, mitochondria, as the main producers of ROS in tissues, play a very important role in this process. Studies of samples from various organs of septic patients and experimental evidence obtained on animals with induced sepsis showed that, as the severity of disease increases, the oxygen consumption in tissues falls [9,10]. Recent data indicate that these processes are related to the disturbances of mitochondrial functions [11-13]. The causes of mitochondrial dysfunction in the systemic inflammatory response syndrome remain unknown.

Microbial metabolites are actively involved in maintaining the homeostasis of a healthy human body and contribute, positively or negatively, to the development of pathological processes. It was found that the level of some microbial metabolites, in particularly phenolic acids, was substantially changed in the blood sera from patients with sepsis [14,15]. In critical states, such as pneumonia associated with artificial lung ventilation and sepsis, a multiple increase in the level of p-hydroxyphenylacetic, phenyllactic, and p-hydroxyphenyllactic acids occur, whereas the amount of phenylacetic and phenylpropionic acids decreases compared with the norm. There is evidence that low molecular weight microbial metabolites of phenolic nature affect the individual enzymes, receptors, and ROS production [16-18]. The pathophysiological role of phenolic acids in the development of sepsis is not clear. In this work, we studied the role of low-molecular-weight phenolic acids of microbial origin in the dysfunction of mitochondria and neutrophils. It was found that phenolic acids exerting pro-oxidant effects on mitochondria inhibited ROS formation by neutrophils. The species-specific production of these metabolites by the anaerobic human microflora and aerobic pathogenic bacteria was determined. The results obtained permit us to consider phenolic acids of microbial origin as biomarkers in the progress of sepsis.

## Methods

### Microorganisms

The ability of bacteria to produce phenolic acids in vitro was studied on isolated microbial strains, which were divided into two groups depending on cultivation conditions:

(1) Anaerobic bacteria (species *Bifidobacteria, Lactobacteria, Bacteroides, Eubacteria,* and *Clostridia*) as representatives of the microbiota of a healthy human. Cultivation was carried out under anaerobic conditions on Schaedler medium [19];

(2) Facultatively aerobic bacteria (species *Staphylococcus, Enterococcus, Escherichia,* and *Klebsiella*) and aerobic bacteria (non fermenting gram-negative species *Pseudomonas* and *Acinetobacter*), whose portion in ill patients increases with the alteration of the gut flora. These bacteria are known as the main causing agents of purulent complications, bacteremia, and sepsis. Cultivation was carried out under aerobic conditions in tryptone soya broth (HIMEDIA) [14].

### Gas chromato-mass spectrometry (GC-MS) analysis

Samples of bacterial cultures were centrifuged for 15 min at 800 *g*, and an internal standard for GC-MS analysis (10 μl of ethanol solution containing 400 ng D_5_-benzoic acid) was introduced into the supernatant (1 ml). Then, each sample was treated with diethyl ether (2 x 1 ml) at pH 2, and the ether extract was evaporated to dryness at 40^о^C. For obtaining trimethylsilyl (TMS) derivatives of the phenolic acids, the residue was treated with 20 μl of N,O-bis(trimethylsilyl)trifluoroacetamide (BSTFA) (Fluka) at 80°C for 15 min. The resulting sample was dissolved in hexane (80 μl) and analyzed by the GC-MS method. Control samples were subjected to analogous treatment. Compounds in samples of microbial cultures were analyzed using an Agilent 6890/5973 gas chromato-mass spectrometer (Agilent Technologies, USA) in the full scan mode [19]. The components were separated by chromatography on a HP5MS quartz capillary column. Mass spectrometry data for the identification of the compounds were obtained using the database NIST-02. The content of individual compounds in samples was determined in triplicates. The results were recalculated in μg/ml and were well reproducible. The statistical significance of difference was estimated by the Student’s *t*-tests. The data shown represent the means ± standard error of means (S.E.M.).

### Isolation of mitochondria

The liver and blood samples were taken from male Wistar rats (200–250 g) after decapitation. This study respected the European Union regulations for animal experiments. Mitochondria were isolated from the liver of Wistar rats by the standard procedure of differential centrifugation in medium containing 300 mM sucrose, 1 mM EGTA, and 10 mM Tris–HCl buffer (pH 7.4) [18]. A mitochondrial pellet was washed twice with the isolation medium containing no EGTA, resuspended in the medium of the same composition, and stored on ice. The protein content was determined by the Lowry method.

### Isolation of neutrophils

Peripheral blood from rat was collected into tubes with anticoagulant (11 mg heparin in 4 ml phosphate buffered saline (PBS) containing 136.9 mM NaCl, 2.7 mM KCl, 1.9 mM NaH_2_PO_4_) and neutrophils were isolated by a combination of hypotonic treatment and Ficoll-urografin gradient centrifugation [20,21]. For hypotonic hemolysis heparinized blood was mixed with cold distilled water in a ratio of 1:2 for 20 s and after the restoration of tonicity by twice PBS solution was centrifuged for 5 min at 180x g at 4^o^C. The pellet was washed with PBS and 2 ml of leukocyte suspension was layered on the gradient of Ficoll- urografin 1,077/1,119 (3 ml) and centrifuged for 15 min at 180x g at 4^o^C. A fraction of cells obtained was twofold washed with PBS and resuspended in Hanks solution (pH 7.2). The content of neutrophils in the fraction was no less than 96%.

### Mitochondrial membrane potential and oxygen consumption assay

The rate of oxygen consumption by mitochondria was determined polarographically using an electrode of a closed oxygen meter of the Clark type. The difference of electric potentials on the inner mitochondrial membrane was determined from the distribution of the lipophilic cation of tetraphenylphosphonium (TPP^+^) whose concentration in external medium [TPP^+^_out_ was registered using a TPP^+^-selective electrode [22]. The parameters were simultaneously registered using a multichannel computerized device Record-4 (Russia). The incubation medium contained 120 mM KCl, 2 mM KH_2_PO_4_, 4 mM pyruvate, 4 mM malate, and 10 mM HEPES (pH 7.4). Other experimental conditions are given in figure captions. The data of typical experiments performed no less than in triplicates with different mitochondrial preparations are presented.

### ROS production

The production of ROS in mitochondria was determined by measuring the chemiluminescence of the Cypridina luciferin analog 2-methyl-6-(*p*-methoxyphenyl)-3,7-dihydroimidazo[1,2-a]pyrazine-3-one (MCLA) [23]. MCLA-dependent chemiluminescence was recorded by a СL-100 chemiluminometer (Russia) simultaneously in several cuvettes with samples (250 μl) containing 0.5 mg of mitochondrial protein, 4 mM substrate of oxidation, and 40 μM MCLA. The generation of superoxide anion was induced by 25 μM menadione [24]. The production of ROS by phagocytes was examined by the method of luminol-dependent chemiluminescence (LCL) [20,21]. Measurements were carried out on a Tecan Infinite F200 microplate reader (Austia) in 96-well plates (Greiner CELLSTAR). The effect of compounds being tested was assessed as follows. Neutrophils (150000 cells per sample) were incubated for 5 min at 37^o^C in Hanks medium containing luminol (100 μM) to which a corresponding compound was added (100 and 200 μM). Then, a phorbol 12-myristate 13-acetate (PMA) 0.28 μM solution was added to the suspension of neutrophils, and LCL was recorded. The changes in LCL intensity by the action of the compounds were estimated relative to the control LCL values: I_LCL_ = I _PA+PMA_/I _solvent+PMA_) x 100%, where I_LCL_ is the recorded value of LCL, I _PA+PMA_ is the intensity of LCL of a suspension of neutrophils in the presence of substances being examined and PMA, I_solvent+PMA_ is the intensity of LCL of a suspension of neutrophils in the presence of alcohol (in a volume equivalent to that of phenolic acids) and PMA. The value of LCL of a suspension of neutrophils in the presence of alcohol served as a background value. Statistical processing of the data was carried out using the program MS Excel 2003. The differences were considered significant at P ≤ 0.05.

## Results

**Figure 1:**
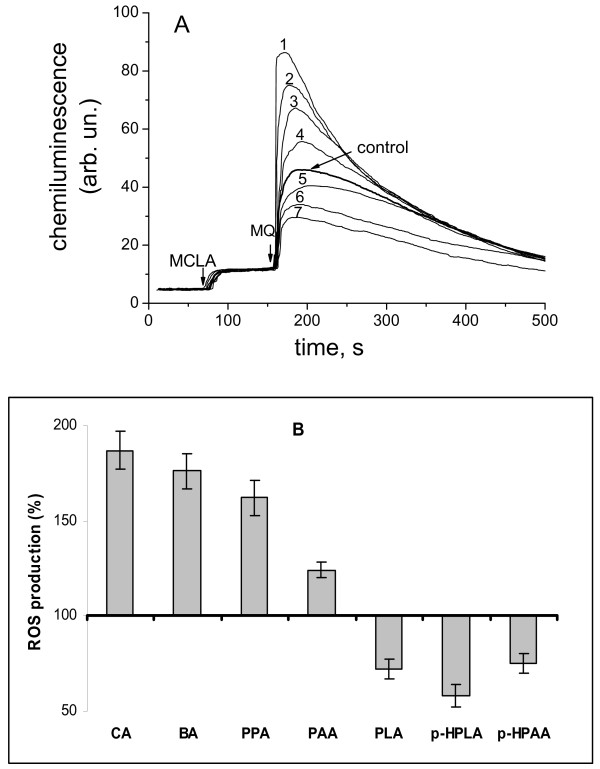
**Effect of phenolic acids on ROS production in mitochondria. A**- Effect of phenolic acids on chemiluminescence of MCLA in mitochondria in the presence of redox-cycler menadione (MQ): 1, cinnamic; 2- benzoic, 3- phenylpropionic, 4- phenylacetic, 5- control, 6- p-hydroxyphenylpropionic, 7- phenyllactic; the concentrations of phenolic acids 100 μM, menadione 25 μM, MCLA 40 μM, pyruvate 4 mM, and rotenone 2.5 μM. **B**- Comparison of the effect of phenolic acids: cinnamic (CA), benzoic (BA), phenylpropionic (PPA), phenylacetic (PAA), *p*-hydroxyphenylpropionic (*p*-HPPA), phenyllactic (PLA), *p*-hydroxyphenyllactic (*p*-HPLA) at a concentration of 100 μM on menadione-activated ROS production. Data are expressed as the means ± SEM of five independent determinations.

The effect of phenolic acids on the menadione-activated ROS production in mitochondria was determined by measuring the chemiluminescence of MCLA (cypridina luciferin analog 2-methyl-6-(*p*-methoxyphenyl)-3,7-dihydroimidazo[1,2-a]pyrazine-3-one). As seen in Figure 1A menadione induced generation of superoxide anion in rat liver mitochondria (control). In the presence of cinnamate, benzoate, and 3-phenylpropionate, the rate of ROS production was substantially higher than in the presence of menadione alone (traces 1–3). A similar effect was observed in the presence of phenylacetate (trace 4), whereas phenyllactate, *p*-hydroxyphenylacetate, and р-hydroxyphenyllactate decreased ROS production compared with the control (traces 5 – 7). Figure 1B summarizes effects of phenolic acids at a concentration of 100 μM on the menadione-activated ROS production.

**Figure 2:**
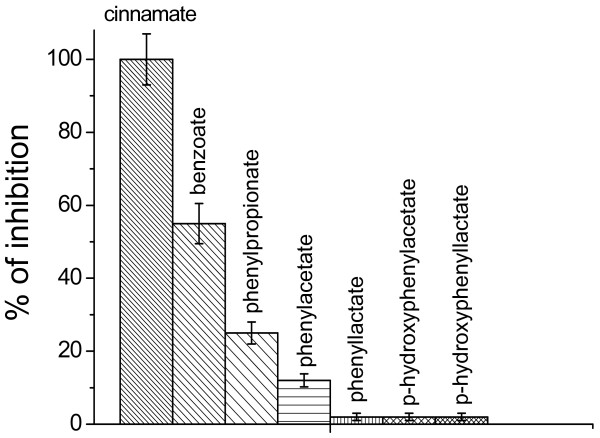
**Effect of phenolic acids of microbial origin on NAD- dependent respiration of mitochondria. **The inhibition of NAD- dependent respiration of mitochondria by cinnamic acid was taken to be 100%. The concentration of phenolic acids was 100 μM. Data are expressed as the means ± SEM of five independent determinations.

**Figure 3:**
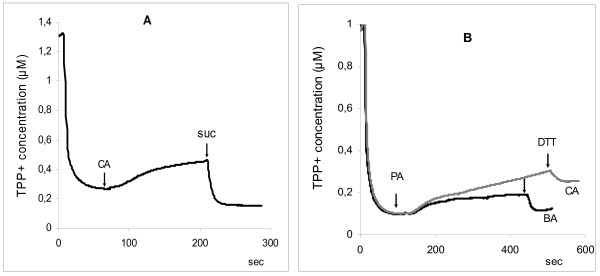
**Effect of cinnamic and benzoic acids on mitochondrial membrane potential. A**- Effect of cinnamic acid (CA) on ΔΨ_m_ upon the oxidation of pyruvate and its elimination by succinate; **B** – Removal of the effects of cinnamic (CA) and benzoic (BA) acids by DTT. The concentrations of phenolic acids were 200 μM, DTT 2 mM, pyruvate 4 mM, and succinate 4 mM.

Similar data were obtained in the study of the effect of the phenolic acids on respiration of mitochondria oxidizing pyruvate, substrate of complex I of respiratory chain. It was found that phenolic acids, which increased of the menadione-activated ROS production, inhibited mitochondrial respiration, whereas with phenyllactate, *p*-hydroxyphenylacetate, and р-hydroxyphenyllactate statistically differences were not observed (Figure 2). Moreover the increase of concentration of cinnamate, benzoate, and phenylacetate (> 100 μM) led to a decrease in the mitochondrial membrane potential upon pyruvate oxidation. The addition of succinate restored the potential to the control level (Figure 3A). Dithiotreitol (DTT) also restored the potential to the control level (Figure 3B). The SH-reagent N-ethylmaleimide (NEM) at concentrations above 250 μM acted similarly, and its effect on the mitochondrial membrane potential was prevented by the preliminary addition of DTT (data not shown).

**Figure 4:**
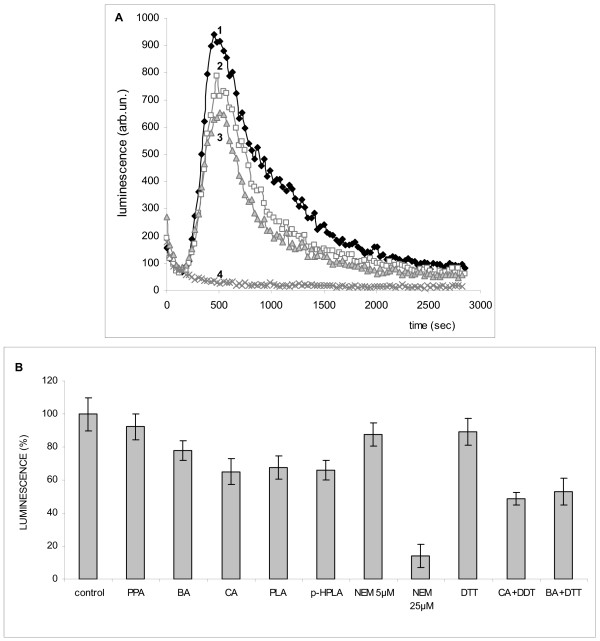
**Effect of phenolic acids of microbial origin and thiol reagents on ROS production in neutrophils.A **– chemiluminescence of luminol in PMA-activated neutrophils, 1- control, 2- benzoic acid, 3- cinnamic acid, 4- NEM, 25 μМ; **B**- effect of the phenolic acids (100 μМ) and thiols reagents (DTT, 1 mM and NEM, 5 and 25 μМ) on ROS production in neutrophils.

All phenolic acids decreased ROS production in PMA-activated neutrophils (Figure 4 A). DTT did not eliminate the effect of cinnamic and benzoic acids on neutrophils, as distinct from mitochondria. Compared with cinnamic and benzoic acids, which acted at concentrations of 100–200 μM, NEM was more effective in inhibiting the ROS production. It produced the inhibitory effect even at a concentration as low as 5 μM and an almost 100% inhibition at a concentration of 25 μM. All other phenolic acids decreased ROS production at different degree (Figure 4 B).

**Figure 5:**
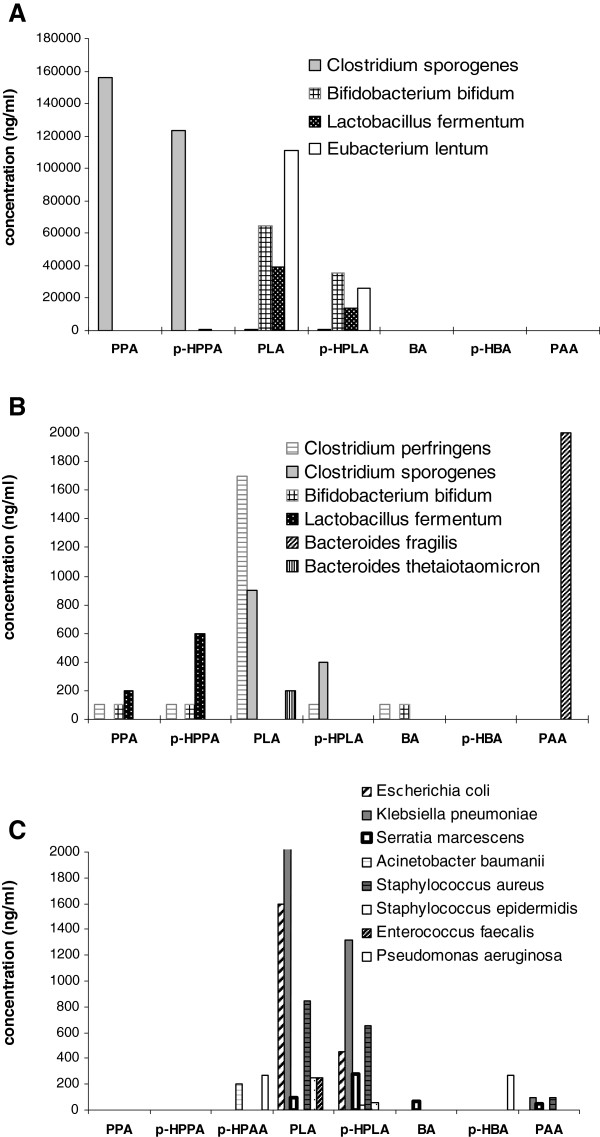
**Species-specific production of phenolic acids by anaerobic human microbiota and pathogenic facultative aerobes. A**- abundant phenolic acids (ng/ml) produced by anaerobic human microbiota; **B** – minor phenolic acids (ng/ml) produced by the same microbiota; **C** - phenolic acids (ng/ml) produced by pathogenic facultative aerobes.

To identify the species-specific production of these phenolic acids by microorganisms, we studied the formation of phenolic metabolites in monocultures by the members of the main groups of the anaerobic human microflora and aerobic pathogenic bacteria by the method of gas chromatography–mass spectrometry. Figure 5 shows the level of phenolic acids produced by anaerobic human microflora (A, B) and aerobic pathogenic bacteria (C). It was found that bifidobacteria and lactobacilli produce in vitro considerable amounts of phenyllactic (64.9 and 35.4 μg/ml, respectively) and *p*-hydroxyphenyllactic acids (39.4 and 13.7 μg/ml, respectively). *Clostridia sporogenes*, as distinct from *C. perfringens*, produced great quantities of phenylpropionic (155.7 μg/ml) and *p*-hydroxyphenylpropionic acids (123.2 μg/ml). Bacteroids produced only phenylacetic acid, and *Eubacterium lentum* formed phenyllactic acid in great amounts (111.2 μg/ml). In aerobic bacteria, the capacity for the production of phenyllactic and *p*-hydroxyphenyllactic acids is hundreds of times lower (Figure 5C). Phenyllactic (0.25–3.5 μg/ml) and *p*-hydroxyphenyllactic (0.45–1.3 μg/ml) acids were produced by *Escherichia coli*, *Klebsiella pneumonia*, *Staphylococcus* spp, and *Enterococcus faecalis*. Non-fermenting gram-negative *Pseudomonas aeruginosa* and *Acinetobacter baumanii*, which normally are absent in the microbiota of the human intestine, did not produce phenyllactic and *p*-hydroxyphenyllactic acids at all. However, they specifically produce *p*-hydroxyphenylpropionic acid, which is absent in the overwhelming majority of bacteria examined. *p*-hydroxybenzoic acid was produced by *Pseudomonas aeruginosa*, and benzoic acid, by *Serratia marcescens*. Cinnamic acid was not detected as a metabolite of these microorganisms when cultivated in monocultures. Thus, these results reveal species-specific production of individual phenolic acids by bacteria.

## Discussion

The results of the study indicate that low-molecular weight phenolic acids of microbial origin can be involved in the regulation of inflammatory syndrome. All phenolic acids had impact on mitochondria and neutrophils, the main producers of ROS in tissues and circulation. It was found that the most effective activators of ROS production in mitochondria are those phenolic acids whose effect is mediated via the interaction with thiol groups. Among these are cinnamic and benzoic acids, the effects of which, similar to the effect of NEM, was abolished by DTT. As it follows from our data, this effect is related to the inhibition of complex I of the respiratory chain. Other phenolic acids, such as phenyllactate and *p*-hydroxyphenyllactate, decreased the ROS production in mitochondria, exhibiting the antioxidant effect.

All phenolic acids decreased the ROS production in neutrophils. This finding agrees well with the earlier reported inhibition of chemiluminescence in human neutrophils in the presence of 3,4-dihydroxyphenylacetic, 3-hydroxyphenylacetic, and other monocyclic phenolic acids generated by the human intestinal microflora [25-28]. The inhibition was related to the scavenging of the superoxide anion. This mechanism may also underlie the action of phenyllactic and *p*-hydroxyphenyllactic acids on mitochondria and neutrophils. On the contrary, cinnamic and benzoic acids do not act as ROS scavengers but exhibit the prooxidant activity, interacting with thiol groups. Presumably, their effect can be explained by the inhibition of NADPH-oxidase. It has been shown earlier that redox processes, among them thiol-dependent, are involved in the mechanism of action of NADPH oxidase inhibitors [28].

Some phenolic acids, in particular phenyllactate and hydroxyphenyllactate, which decrease ROS production in both mitochondria and neutrophils, can have the protecting effect on organs and tissues. They can play a role of natural antioxidants. Recent trends are towards the use of mitochondrial therapy aimed at providing cells and tissues with particular antioxidants. Indeed, in some cases, the application of antioxidants in induced sepsis led to the improvement of oxidative phosphorylation and a partial restoration of hemodynamic parameters and functions of the organism [29,30]. Based on these data, it can be assumed that, when affecting targets in the organism, microbial metabolites can enhance or reduce the inflammatory syndrome. If this is so, the physiological significance of these effects of phenolic acids can be different depending on the stage of the inflammatory syndrome since the response to a septic insult is a dynamic process characterized by distinct differences between the acute and chronic phases of the illness. By inhibiting ROS production in neutrophils, they decrease the immune response of the organism, which can produce a beneficial effect at late stages of sepsis associated with bioenergetic failure and an adverse effect at the early stage of pro-inflammation.

All of these metabolites have been identified in the intestine and blood. The total concentration of phenolic acids in the human intestine exceeds 400 μM [31], in blood it varies from 5.3 μM in the norm to 55 μM in sepsis [15,18]. There is evidence indicating a strong increase in the level of phenylacetate to 3.49 mmol/l in the blood plasma of patients with end-stage renal disease [16]. In the present study we obtained data on the species-specific production of those metabolites that affect the functions of mitochondria and neutrophils. Below primary emphasis in the analysis will be placed on those phenol acids that show the capacity to suppress ROS production by both mitochondria and neutrophils. These are phenyllactic and *p*- hydroxyphenyllactic acids. The results showed that all anaerobes studied (except *Bacteroides fragilis*) are capable of producing these acids, and the greatest quantities (39–111 μg/ml of phenyllactic acid and 26–35 μg/ml of *p*- hydroxyphenyllactic acid) are produced by *Eubacterium lentum, Bifidobacterium bifidum,* and *Lactobacterium fermentum*. It is known that just these gram-positive anaerobic bacteria form a dominant pool of the intestinal microbiota of a healthy human. This fact may point to the biological expediency of the capacity of these bacteria for phenyllactic and *p*- hydroxyphenyllactic acids production as a mechanism of host–bacteria interaction that prevents the development of inflammation in response to the continuous contact of these bacteria with the intestinal mucosa. In septic patients, this physiological role of anaerobes is lost since the vital activity of bifido-, lato-, and eubacteria in the ill organism is suppressed, so that they are almost entirely eliminated from the intestine. In aerobic bacteria, phenyllactic and *p*- hydroxyphenyllactic acids were produced only by those bacteria that are constantly present in the biocenosis of the human (*Escherichia coli*, *Klebsiella pneumonia*, *Staphylococcus* spp, *Enterococcus faecalis*). Non-fermenting gram-negative *Pseudomonas aeruginosa* and *Acinetobacter baumanii*, which normally are absent in the microbiota of the human intestine, did not produce phenyllactic and *p*- hydroxyphenyllactic acids at all. However, these microorganisms specifically produce *p*-hydroxyphenylacetic acid, which is absent in the overwhelming majority of bacteria examined. In our experiments, *p*-hydroxyphenylacetic acid showed the capacity to inhibit the ROS production in neutrophils. During the development of bacteremias and purulent foci of infection associated with *Pseudomonas aeruginosa* and *Acinetobacter baumanii*, their metabolite *p*-hydroxyphenylacetic acid can directly enter the systemic blood flow and inhibit the phagocytic activity of neutrophils.

The production of other metabolites is more species-specific. Among metabolites increasing the ROS production in mitochondria, the production of phenylacetic acid was observed in *Bacteroides fragilis*, of phenylpropionic acid, in *Clostridium sporogenes*. Benzoic acid was found in minor amounts in *Serratia marcescens*. Cinnamic acid was absent in isolated bacterial strains; however, the fact of its microbial origin was documented in other studies where benzoic and cinnamic acids were detected in plasma and fecal water from human volunteers [31]. Cinnamic acid is a product of phenylalanine ammonia-lyase, a ubiquitous higher-plant enzyme that catalyzes the nonoxidative deamination of phenylalanine to cinnamic acid. This enzyme was encountered only in a few bacteria, where it is involved in benzoyl-CoA biosynthesis, namely in “*Streptomyces maritimus*” and *Sorangium cellulosum*, as well as in the biosynthesis of cinnamamide in *Streptomyces verticillatus*[32-34].

It is known that the inflammatory response is characterized by a massive increase in the production of reactive oxygen species, nitric oxide, and inflammatory cytokines. These pathologic factors have a great impact on mitochondrial functions in sepsis [5]. As distinct from pathogen-associated molecular patterns such as bacterial lipopolysaccharides and lipoteichoic acids, which are recognized by immune cells and initiate an acute response to pathogens [4], microbial phenolic acids may be assumed to regulate the magnitude of this response. A source of phenolic acids in the organism is the anaerobic degradation of aromatic amino acids and polyphenols by microflora. Consequently, nutritional or medicinal plant foods may influence the concentration of gut microbial-derived low-molecular phenolic acids. In humans, the antioxidants such as 4-hydroxyphenylacetic, 4-hydroxyphenylpropionic, and 3,4-dihydroxyphenylacetic acids were identified as the metabolites of quercetin, catechin, and other polyphenols [35,36]. As follows from our data, phenolic acids act on targets that produce ROS in the organism. Some phenolic acids, which decrease ROS production in both mitochondria and neutrophils, can play a role of natural antioxidants. By affecting neutrophils, they retard the immune response, whereas while acting on mitochondria, they prevent the development of multiple organ failure or reduce it.

## Conclusions

According to the data of clinical examination, the high levels of phenyllactic and *p*- hydroxyphenyllactate acids in blood correlate with the development of sepsis and the risk of lethal outcome. There is evidence for the microbial origin of these phenolic acids in human blood. The suppression of ROS production by phenyllactic, *p*- hydroxyphenyllactic acids, and *p*-hydroxyphenylacetic acid in mitochondria and neutrophils can substantially contribute to the course of local infections and sepsis. The results obtained are particularly urgent in view of the fact of their production by *Klebsiella pneumonia, Escherichia coli, Pseudomonas aeruginosa,* and *Acinetobacter baumanii*, which are the most frequent causing agents of hospital infectious complications and sepsis.

## Abbreviations

SIRS: Systemic inflammatory response syndrome; ROS: Reactive oxygen species; NO: Nitric oxide; GC-MS: Gas chromato-mass spectrometry; PBS: Phosphate buffered saline; TPP: Tetraphenylphosphonium; MCLA: 2-Methyl-6-(*p*-methoxyphenyl)-3,7-dihydroimidazo[1,2-a]pyrazine-3-one; LCL: Luminol-dependent chemiluminescence; PMA: Phorbol 12-myristate 13-acetate; DTT: Dithiotreitol; NEM: N-ethylmaleimide.

## Competing interests

The authors declare that they have no competing interests.

## Authors’ contributions

IB, AO, VS, VT and NF carried out experimental work and analyzed data. NB, NF and VT designed the study, coordinated the experiments, analyzed data and wrote the manuscript. All authors read and approved the final manuscript.
